# Reducing Emergency Department Strain Through Usable Wearables: A Proof-of-Concept for Multi-sensor Remote Patient Monitoring

**DOI:** 10.7759/cureus.95053

**Published:** 2025-10-21

**Authors:** Drin Rrmoku

**Affiliations:** 1 Independent Electrical Engineer, Prishtina, ALB

**Keywords:** battery efficiency, bluetooth health tech, emergency department overcrowding, fall detection, remote patient monitoring, vital signs sensors, wearable devices, wireless charging

## Abstract

Rising emergency department (ED) cases are straining hospital resources, compromising care quality, and elevating mortality risks. Remote patient monitoring via wearable devices offers a pathway to alleviate this burden, yet many are hindered by short battery life, discomfort, and limited clinical utility. This proof-of-concept describes a compact, wrist-worn wearable for continuous remote health monitoring. It integrates multi-sensors for heart rate, body temperature, blood oxygen saturation, and accelerometer-based motion/fall detection. Data streams via Bluetooth to a smartphone app, triggering alerts for vital sign deviations or falls. Usability is enhanced with wireless charging, a water-resistant enclosure, and a capacitive wear-detection mechanism to optimize battery efficiency. We present full schematics and a 3D model demonstrating seamless integration of sensors, a Bluetooth module, and power management into a low-cost, ergonomic device. These features enable multifunctional, daily-use monitoring, addressing key barriers to adoption. This usable wearable design supports extramural patient care through vital tracking, fall alerts, and practical innovations, potentially reducing ED overcrowding and enabling timely interventions for at-risk groups. Though unprototyped and unvalidated, it underscores the feasibility of scalable remote solutions. Future efforts will prioritize fabrication, accuracy testing, and clinical pilots.

## Introduction

Over the past few years, emergency departments (ED) have experienced overcrowding rates exceeding 80% in urban hospitals, with significant strain reported in regions such as North America and Europe [1,2]. Moreover, the healthcare system's underpreparedness for an aging population has exacerbated resource strains, diminished care quality, and increased mortality rates [2]. A persistent shortage of qualified professionals, including physicians, nurses, pharmacists, clinical social workers, and technicians, further hampers the system's capacity to meet patient demands [3].

Remote patient monitoring via smart wearable devices has emerged as a promising countermeasure. These technologies enhance care quality, ease system pressures, and lighten healthcare professionals' workloads. Capable of tracking diverse biometrics such as heart rate, oxygen saturation, and activity levels [4], wearables are increasingly woven into daily life, enabling broad-scale monitoring of diverse populations. In fact, remote monitoring has been linked to shorter hospital stays in select cases, including cardiac arrhythmia detection, post-COVID-19 patient follow-up, and elderly fall-risk monitoring [5,6].

As mature medical tools, wearables embody five core attributes: (1) wireless mobility, (2) interactivity and intelligence, (3) sustainability and durability, (4) simple operation and miniaturization, and (5) wearability and portability. They span four key applications: (1) health and safety monitoring, (2) chronic disease management, (3) disease diagnosis and treatment, and (4) rehabilitation [6,7].

Yet, despite their potential, wearables face notable barriers. Short battery life curtails continuous monitoring [8], while bulky designs and skin irritation undermine comfort and adherence [9]. Many also fall short on clinical accuracy, eroding trust for decision-making [10]. Moreover, privacy and data security issues demand stronger protections for healthcare integration [11].

This paper introduces a proof-of-concept for a compact, wrist-worn wearable tailored to continuous remote health monitoring to reduce ED strain through enhanced usability to bridge these gaps. The device fuses heart rate, body temperature, and blood oxygen saturation sensors with a digital accelerometer for motion tracking and fall detection. Bluetooth streams data to a smartphone app for real-time visualization and automated anomaly alerts. Usability shines via wireless charging (bypassing cumbersome ports) and a water-resistant enclosure for seamless daily integration. By prioritizing low cost, practicality, and patient-centered design, this solution promises reliable, accessible monitoring for patients and providers alike.

## Technical report

The system's brain is the NINA-B306 controller, a low-power Bluetooth-enabled microcontroller responsible for coordinating sensor data acquisition, processing, and wireless transmission to a smartphone application. The controller was selected for its combination of compact size, energy efficiency, and integrated Bluetooth low energy (BLE) functionality, which supports seamless communication with mobile devices. Programming pads were included in the design to allow firmware development and updates, enabling flexibility for future modifications or feature expansion.

The MAX30102EFD+T is incorporated to monitor cardiovascular status, delivering both blood oxygen saturation and pulse rate. A MAX30205 digital temperature sensor was integrated to enable accurate body temperature monitoring, critical for detecting early signs of infection or fever. To capture physical activity and support fall detection, an ADXL345 digital accelerometer was included. A capacitive touch sensor was added to serve as a wear-detection mechanism. When skin contact is detected, the sensor signals the NINA-B306 controller, activating the other integrated circuits. This approach minimizes unnecessary power consumption by ensuring that the sensing modules are only active when the device is worn, thereby extending battery life and improving overall system efficiency.

The device is powered by a small rechargeable lithium battery. It is integrated into the design and sealed inside the enclosure to ensure safety and durability. To enhance practicality, the design incorporates a BQ51050B wireless charging controller and associated charging coil, eliminating the need for external connectors prone to mechanical wear and potential water ingress.

**Figure 1:**
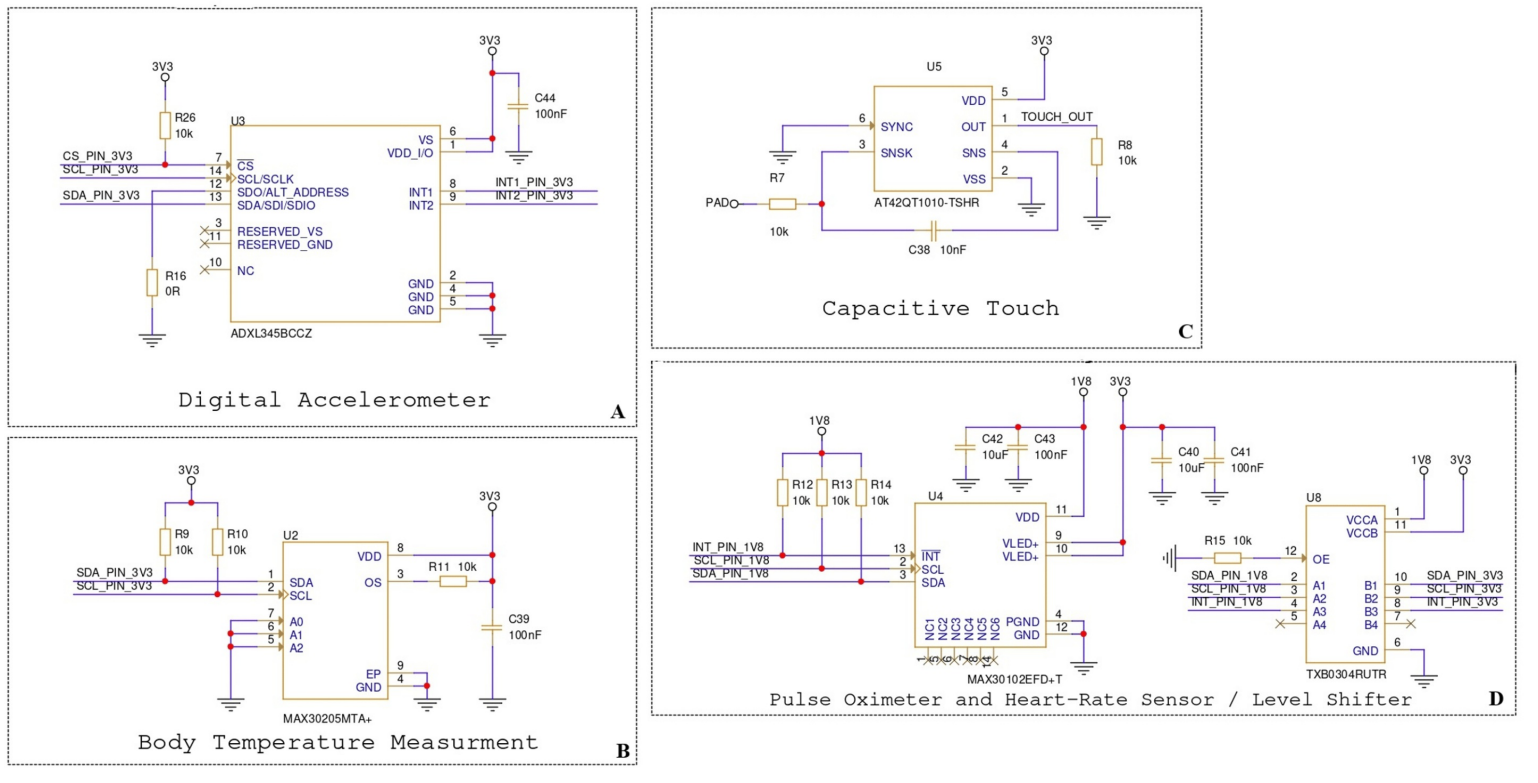
Schematic diagrams of the sensor and interface subsystems (A) Digital accelerometer circuit using the ADXL345BCCZ for motion and fall detection. (B) Body temperature measurement circuit based on the MAX30205MTA+ digital temperature sensor. (C) Capacitive touch input circuit utilizing the AT42QT1010-TSHR for user interaction. (D) Pulse oximeter and heart-rate sensor (MAX30102EFD+T) with TXB0304 level shifter for signal compatibility. HR: heart rate, SpO₂: oxygen saturation, VCC: supply voltage, I²C: inter-integrated circuit, °C: temperature, bpm: heart rate, g: acceleration, V: voltage Image Credit: Designed by the author

The complete schematic and 3D printed circuit board (PCB) model developed from these hardware components are presented in the Results section to demonstrate integration and feasibility. Figure 1 shows the sensor subsystem schematic of the proposed wearable device, integrating the MAX30102 pulse oximeter and heart-rate sensor, MAX30205 digital temperature sensor, ADXL345 accelerometer, and capacitive touch sensor.

**Figure 2:**
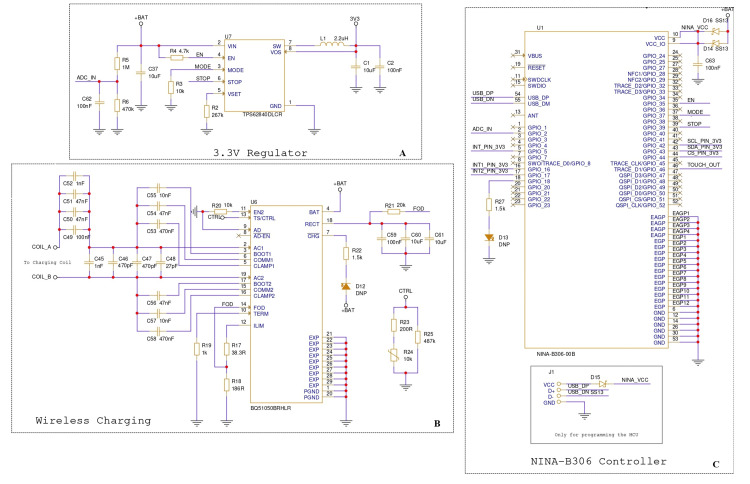
Schematic diagrams of the power regulation and control subsystems (A) 3.3V regulator circuit based on the TPS62840DLCR, responsible for supplying stable power to the system. (B) Wireless charging circuit utilizing the BQ51050BHLR for inductive power transfer and battery management. (C) NINA-B306 microcontroller schematic showing connectivity, input/output pins, and programming pins. BLE: Bluetooth low energy, IC: integrated circuit, VCC: supply voltage, GND: ground, V: voltage, mA: current, W: power Image Credit: Designed by the author

Figure 2 illustrates the power management and NINA Bluetooth module subsystem schematic. The design integrates a TPS62840-based 3.3V regulator for stable power delivery, a BQ51050B wireless charging controller for contactless charging, and the NINA-B306 microcontroller, which manages sensor data acquisition and Bluetooth communication.

**Figure 3:**
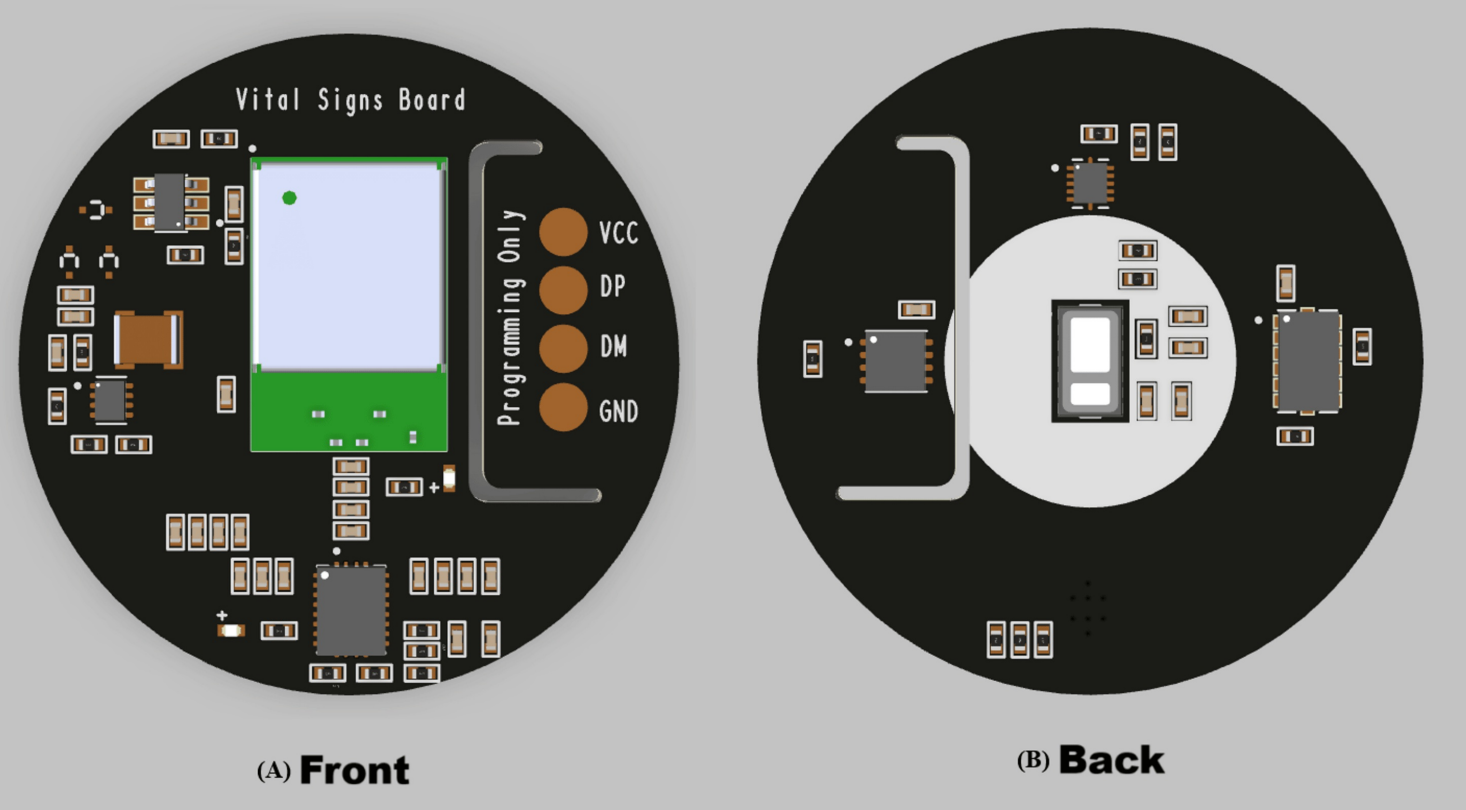
3D model of the PCB (A) Front view showing the NINA Bluetooth module, wireless charging IC, programming pins, voltage regulator, and capacitive touch sensor. (B) Back view showing the level shifter, digital accelerometer, and blood oxygen/heart rate sensor. BLE: Bluetooth low energy, IC: integrated circuit, PCB: printed circuit board, HR: heart rate, SpO₂: oxygen saturation, 3D: three-dimensional, mm: dimensions, V: voltage Image Credit: Designed by the author

Figure 3 presents the 3D model of the PCB with complementary components, shown in front (left) and back (right) views. The circular form factor was chosen for integration into a wrist-worn enclosure. Key components, including the NINA-B306 controller and supporting circuitry, are positioned to optimize space utilization and maintain signal integrity.

**Figure 4:**
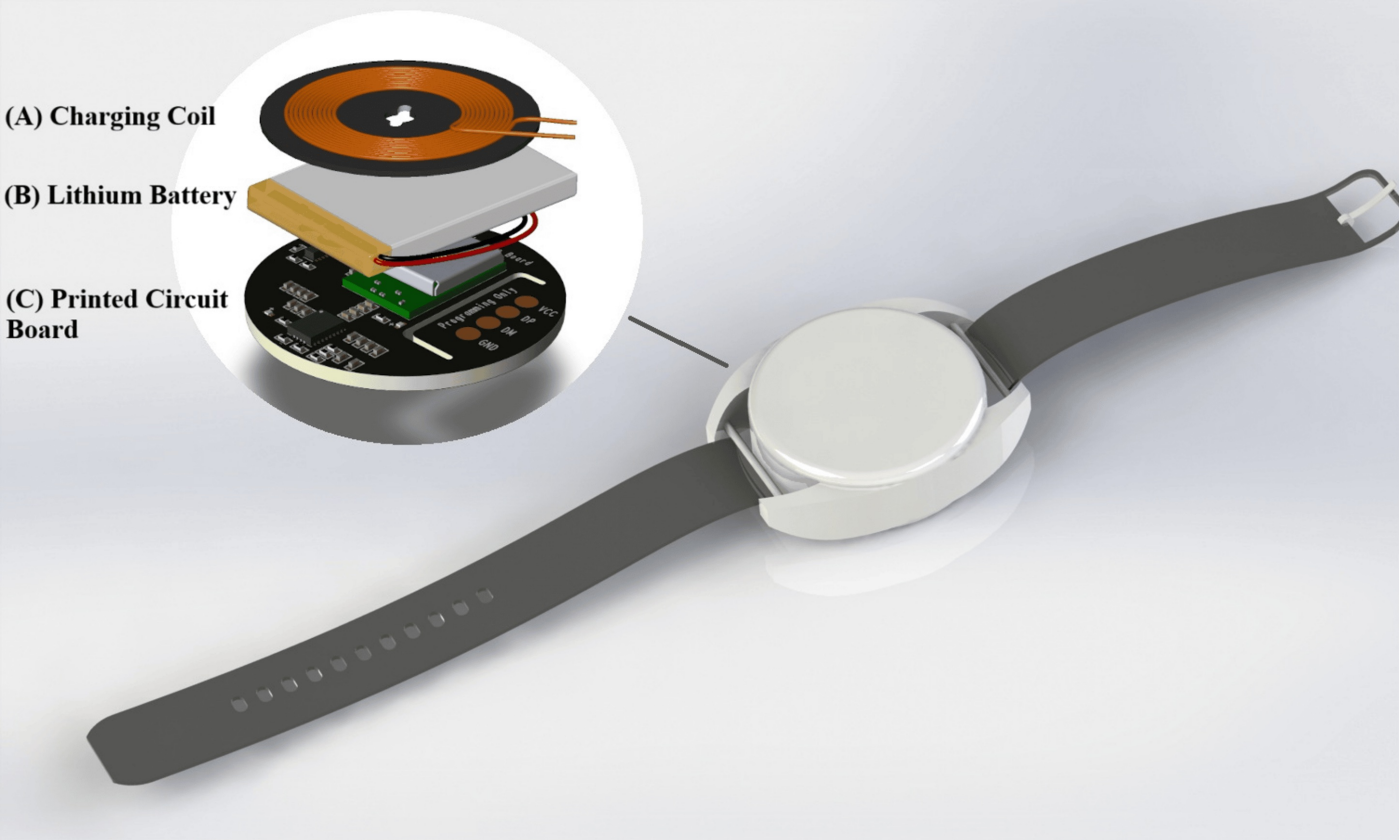
Exploded 3D model of the wearable device enclosure with key internal components (A) Charging coil for wireless power transfer. (B) A lithium battery provides energy storage. (C) The custom PCB integrates vital signs monitoring components, including sensors, a microcontroller, and a wireless charging circuit. PCB: printed circuit board, IC: integrated circuit. 3D: three-dimensional Image Credit: PCB and enclosure models designed by the author. The charging coil and battery 3D models were adapted from GrabCAD (“Qi Wireless Charging Coil Ø44mm” by Andrew Ives (https://grabcad.com/library/qi-wireless-charging-coil-o44mm-1) and “523450 Li-Iron Battery 1000mAh 3.7Wh DC4.2V by Dhruv Rudani (https://grabcad.com/library/523450-li-iron-battery-1000mah-3-7wh-dc4-2v-1).”

The wearable device was developed in a compact wrist-worn form factor designed for comfort and long-term use, as shown in Figure 4. Its internal architecture comprises a wireless charging coil, a lithium battery, and a PCB containing the sensing and control electronics. This configuration enables continuous monitoring of basic health parameters, such as body temperature and heart rate, while ensuring patient convenience through cable-free charging and a lightweight design. The exploded view highlights the efficient arrangement of components within the enclosure, demonstrating how the device balances functionality with comfort and safety for everyday health monitoring.

Table 1 summarizes the core hardware components, highlighting their role in achieving a multifunctional, user-friendly system with estimated costs under $40/unit.

**Table 1:** Hardware components

Component	Function	Key specifications	Design benefit
NINA-B306	Microcontroller/BLE	Low-power (1.5 mA active), 10 x 10 mm	Efficient data processing and wireless tx to app
MAX30102	Heart rate and SpO₂ sensor	±2% accuracy, I2C interface	Reliable cardiovascular monitoring in compact form
MAX30205	Temperature sensor	±0.1°C accuracy, digital output	Enables activity tracking and automated fall alerts
ADXL345	Accelerometer for motion/fall	3-axis, 2-16 g range, low-power	User-friendly, no ports
BQ51050B	Wireless charging controller	Qi-compatible, up to 5 W	Hassle-free recharging, no ports
Capacitive touch	Wear-detection	Low-power activation trigger	Optimizes battery by idling when off-wrist

This configuration yields an estimated >7 days of battery life in typical low-activity scenarios (based on 100 mAh LiPo and ~0.5 mA average draw across sensors and BLE, with wear detection minimizing idle consumption). The proposed wearable device addresses several limitations observed in remote patient monitoring systems. The device reduces unnecessary power consumption by incorporating a capacitive touch wear-detection mechanism. It extends operational time, helping to overcome the short battery life that has limited the clinical adoption of many wearables. This ensures that continuous monitoring can be maintained reliably, which is especially important for patients at risk of sudden deterioration.

Comfort and usability were also prioritized in the design. The compact wrist-worn form factor, sealed rechargeable battery, and wireless charging capability eliminate the need for bulky connectors or frequent device handling. These features make the device more acceptable for patients' daily use and reduce the burden on caregivers and healthcare workers, who often face difficulties maintaining and troubleshooting patient equipment.

Finally, the system supports integrating digital health platforms through Bluetooth communication with smartphones. This enables remote transmission of vital signs and automated alerts, allowing healthcare providers to monitor patients without requiring constant in-person contact. Such functionality directly eases the workload of healthcare professionals, reduces unnecessary hospital visits, and may help lower rates of ED overcrowding.

According to component datasheets and preliminary bench evaluations, the MAX30102 and MAX30205 modules offer typical accuracies of ±2% (SpO₂) and ±0.1°C (temperature), respectively. The accelerometer (ADXL345) provides 10-bit resolution across a ±2 g to ±16 g range, enabling reliable motion and fall detection. During periodic sampling with Bluetooth active, the estimated system current draw of ~0.5 mA supports a projected >7-day runtime under low-activity conditions.

## Discussion

ED overcrowding poses a growing threat to patient outcomes and staff well-being, necessitating innovative strategies for relief [12]. Wearable monitoring devices offer a compelling approach, yet consumer-grade options like fitness trackers and smartwatches are hampered by short battery life, suboptimal accuracy, and poor healthcare integration [8-10]. The proposed design mitigates these barriers through targeted features: a capacitive wear-detection mechanism for power efficiency, wireless charging for seamless use, and a sealed rechargeable battery for durability, potentially enabling more than 7 days of runtime in low-activity scenarios.

From a clinical standpoint, these enhancements directly alleviate ED pressures. Wrist-worn remote monitoring facilitates early interventions, curbs non-essential visits, and reassures caregivers, extending care beyond hospital confines. Amid persistent resource strains and elevated mortality [1,2], such practical solutions could substantially reduce overcrowding and bolster provider efficiency.

That said, this proof-of-concept carries key limitations. In the absence of fabrication or clinical trials, aspects like real-world battery endurance, sensor precision, and data fidelity remain untested. Moreover, data security and privacy, which are pivotal hurdles in digital health [11], are not yet addressed, necessitating HIPAA-aligned safeguards in future iterations. Nonetheless, this conceptual blueprint illustrates how low-cost (<$40) innovations in wear detection, charging, and ergonomics can surmount adoption obstacles. It signals the transformative potential of refined wearables in easing global healthcare burdens, paving the way for prototyping, validation, and deployment.

## Conclusions

This proof-of-concept demonstrates a wrist-worn wearable capable of continuous remote health monitoring by integrating vital-sign sensors and accelerometer-based fall detection. The presented data confirm the feasibility of key subsystems, including wear-triggered activation, low-power wireless communication, and sealed battery operation exceeding seven days under low-activity use. These findings substantiate the device’s potential to reduce ED strain, support proactive interventions, and enable seamless provider oversight. While unprototyped at full scale, the current results establish a foundation for future work focused on complete system prototyping, accuracy benchmarking against clinical standards, and pilot testing among at-risk populations to validate real-world performance.
